# Prevalencia del miedo a caer y factores asociados en personas mayores que viven en la comunidad

**DOI:** 10.1016/j.aprim.2020.11.003

**Published:** 2021-01-11

**Authors:** Nuria Alcolea-Ruiz, Sonia Alcolea-Ruiz, Francisco Esteban-Paredes, Milagros Beamud-Lagos, María Teresa Villar-Espejo, Francisco Javier Pérez-Rivas

**Affiliations:** a Centro de Salud Sector 3 (Getafe), Gerencia Asistencial de Atención Primaria del Servicio Madrileño de Salud, Madrid, España; b Hospital La Paz, Madrid, España; c Centro de Salud El Greco (Getafe), Gerencia Asistencial de Atención Primaria del Servicio Madrileño de Salud, Madrid, España; d Centro de Salud Paseo Imperial, Gerencia Asistencial de Atención Primaria del Servicio Madrileño de Salud, Madrid, España; e Departamento de Enfermería, Facultad de Enfermería, Fisioterapia y Podología, Universidad Complutense de Madrid, Madrid, España; f Centro de Salud Reyes Magos (Alcalá de Henares), Gerencia Asistencial de Atención Primaria del Servicio Madrileño de Salud, Madrid, España

**Keywords:** Caídas, Miedo, Persona mayor, Prevalencia, Falls, Fear, Elderly, Prevalence

## Abstract

**Objetivo:**

El objetivo general del estudio es conocer la prevalencia de la preocupación a caer en personas mayores, independientes para la deambulación, que viven en la comunidad, según la versión reducida del FES-I y los factores asociados que influyen en esta preocupación.

**Diseño:**

Estudio descriptivo transversal.

**Emplazamiento:**

Centro de Salud El Greco (Getafe), Gerencia Asistencial de Atención Primaria de Madrid.

**Participantes:**

Ciento ochenta y nueve pacientes ≥ 70 años independientes o con dependencia funcional leve-moderada (índice de Barthel ≥ 60) e independientes para la deambulación (camina 45 min sin ayuda o con bastón). El estudio se ofreció a 328 personas: 217 aceptaron y rechazaron 111.

**Mediciones principales:**

La variable dependiente, miedo a caerse (MC), fue evaluada mediante el cuestionario *Short FES-I*, considerando como punto de corte para el cribado positivo del MC una puntuación ≥ 11. Como variables independientes se consideraron: índice de Barthel, escala Downton, prueba de fragilidad *Short Physical Performance Battery* (SPPB), caídas en el último año, lesiones asociadas a las caídas, tiempo desde la última caída, déficit sensorial, uso de dispositivos de la marcha, comorbilidad y tratamiento farmacológico.

**Resultados:**

La prevalencia del MC fue del 42,9% (IC 95%: 35,5-50,2). Los factores asociados al MC en el análisis multivariante final fueron: sexo femenino, vivir solo, alto riesgo de caídas, presencia de fragilidad, uso de hipotensores y lesiones asociadas a caídas previas.

**Conclusiones:**

La prevalencia del miedo a caer en personas mayores es elevada. Los profesionales de atención primaria deben sistematizar el cribado de este problema de salud, priorizando especialmente en las personas que presentan los siguientes factores de riesgo: ser mujer, vivir solo, tener una puntuación baja en el SPPB (como indicador de fragilidad) o presentar un elevado riesgo de caídas.

## Abstract (en)

**Aim:**

To know, in the population over 70, independent for walking, the prevalence of the concern to fall according to the short version of the Short Falls Efficacy Scale-International (FES-I) questionnaire, in old people living in the community and their associated factors.

**Location:**

Centro de Salud El Greco, Getafe, Madrid, Spain.

**Participants:**

189 patients ≥ 70 years with a Barthel ≥ 60, independent for walking (walk 45 minutes without help or with a cane). The study was offered to a total of 328 people, of these accepted 217 and rejected 111.

**Main measurements:**

The dependent variable, fear of falling (FOF), was evaluated by means of the short FES-I questionnaire, considering as a cut-off point for the positive screening of the MC a score ≥ 11. As independent variables we considered: Barthel index, Downton scale, the Short Physical Performance Battery (SPPB) fragility test, falls in the last year, injuries associated with falls, time since the last fall, sensory deficit, use of gait devices, comorbidity and pharmacological treatment.

**Results:**

The prevalence of FOF was 42.9% (95% CI: 35.5-50.2). The factors associated with FOF in the final multivariate analysis were: female sex, living alone, high risk of falls, presence of frailty (SPPB ≤ 9), use of hypotensive drugs, and injuries associated with previous falls.

**Conclusions:**

The prevalence of FOF in older people is high. Primary Care professionals should systematize the screening of this health problem, prioritizing especially in people who present the following risk factors: being a woman, living alone, having a low score on the SPPB (as an indicator of frailty) or presenting a high risk of falls.

## Introducción

El miedo a caerse (MC), en inglés *fear of falling* (FOF), es «la pérdida de confianza en sí mismo para evitar las caídas durante la realización de actividades esenciales y relativamente no peligrosas, que llevan al individuo a evitar actividades que es capaz de realizar»^1^. Sus implicaciones son especialmente graves debido a que este temor incrementa el riesgo de caídas^2^, ^3^, ^4^ y provoca un deterioro de la capacidad funcional en personas mayores^5^, ^6^, ^7^, ^8^, ^9^. Además, incrementa el riesgo de fragilidad, dependencia y muerte, afectando de un modo integral (física, social y psicológicamente) a las personas con más preocupación por caer^10^, ^11^, ^12^.

Existen muchos instrumentos para medir la preocupación por caerse. El método más utilizado es el cuestionario *Falls Efficacy Scale-International* (FES-I)^13^. Es una escala basada en la teoría de Bandura de la autoeficacia que mide el nivel de confianza demostrado por el paciente para realizar actividades básicas de la vida diaria (ABVD), incluyendo actividades que se realizan fuera del hogar^14^. Kempen et al.^15^ desarrollaron una versión reducida, la *Short FES-I*, con 7 preguntas y un rango de puntuación entre 7 y 28, que comparada con la versión larga demostró una consistencia interna bastante fiable (α-Cronbach = 0,92). Esta versión reducida del cuestionario ha sido validada en español para pacientes mayores por Araya et al.^16^.

El MC es común entre las personas mayores que viven en la comunidad, y su prevalencia oscila en España entre el 41,5 y el 49,4%^7^, ^17^, ^18^. Para la estimación de estas prevalencias, en los estudios de Lavedán et al.^7^ y Parraga et al.^17^ utilizaron una pregunta directa sobre el MC (sí/no o mucho/poco), mientras que Moles et al.^18^ utilizaron la escala de FES-I completa. No se ha realizado ningún estudio en España que haya utilizado la versión reducida del FES-I para evaluar la prevalencia.

En cuanto a los factores que influyen en el desarrollo de la preocupación por caerse, diversos estudios realizados en contextos muy diversos y con distintos tipos de pacientes han identificado como posibles factores de riesgo: edad avanzada, sexo femenino, caídas previas, vivir solo, depresión, ansiedad, trastornos de la marcha y el equilibrio, trastornos del sueño, dolor crónico, tratamiento farmacológico, comorbilidad, déficits sensoriales y calidad de vida^17^, ^18^, ^19^, ^20^, ^21^, ^22^, ^23^.

El objetivo general del estudio es conocer la prevalencia de la preocupación a caer en personas mayores, independientes para la deambulación, que viven en la comunidad, según la versión reducida del FES-I, y los factores asociados que influyen en esta preocupación.

## Material y métodos

### Diseño y población de estudio

Se trata de un estudio descriptivo transversal realizado en el Centro de Salud El Greco (Getafe) de la Gerencia Asistencial de Atención Primaria de Madrid. La población de estudio fueron las personas mayores de 70 años que cumplían los criterios de selección y acudieron al centro de salud entre los meses de abril y junio de 2018.

### Criterios de selección

Con el objetivo de captar a los pacientes en una situación de pre-fragilidad, en los que las medidas de prevención precoz pudieran ser más efectivas a la hora de evitar el inicio de la espiral de deterioro y limitaciones que se producen por el MC, se incluyeron en el estudio a las personas que cumplían los siguientes criterios:

• Nacidas antes del 1 de enero de 1948.
• Independientes para las ABVD o que presentaban dependencia funcional leve-moderada (Índice de Barthel ≥ 60).
• Independientes para la deambulación (caminan 45 min sin ayuda o con bastón).
• Sin necesidad de cuidados paliativos y que no presentaban en el momento de la captación ningún proceso agudo que les produjera disconfort: fiebre, dolor, malestar general….
• Comprendieron el consentimiento informado y los procedimientos que se iban a realizar en el estudio, y su participación no supuso ningún riesgo para su enfermedad o situación funcional.

### Tamaño muestral

Considerando una prevalencia estimada del temor a caer del 40%^7^, ^17^, ^18^ y asumiendo una Zbeta de 0,80 y una Zalfa de 0,95, con una precisión del 7%, se consideró que el tamaño muestral necesario era de 189 individuos.

### Muestreo y reclutamiento

Se llevó a cabo un muestreo consecutivo sobre la agenda de los pacientes citados en las consultas de 5 enfermeras del Centro de Salud El Greco cada día, hasta alcanzar el tamaño muestral requerido. Cada enfermera incluyó al primer y quinto paciente que cumplía los criterios de selección, en un tramo horario definido. Los pacientes que aceptaron fueron derivados a una agenda creada para la realización del estudio. Dicha agenda estuvo formada por citas de 20 min. En los casos en que los pacientes eran captados en un turno diferente al de la agenda, se programaba una cita. El estudio se llevó a cabo entre los meses de abril y junio de 2018. Se realizó un estudio piloto de una semana para comprobar la adecuación de los ítems de valoración y la coordinación entre profesionales. Todas las entrevistas fueron realizadas por un único profesional (investigadora del proyecto).

### Variables de estudio

La variable dependiente, el MC, fue evaluada mediante el cuestionario Short FES-I, considerando como punto de corte para el cribado positivo del MC una puntuación mayor o igual a 11^24^.

Respecto a las variables independientes, se clasificaron en: variables sociodemográficas (edad, sexo, convivencia); funcionales: escala de Downton (indica riesgo de caídas si ≥ 3); índice de Barthel (mide la capacidad de realizar ABVD) y el *Short Physical Performance Battery* (SPPB) como indicador de fragilidad si su puntuación es menor a 10^25^, y variables clínicas (caídas en el último año, lesiones asociadas a las caídas, tiempo desde la última caída, índice de masa corporal [IMC], índice de comorbilidad de Charlson [siendo positivo en caso de 3 o más patologías], uso de gafas, audífono y/o dispositivo para la marcha, uso de medicación [hipotensora, benzodiacepinas, sedantes, antidepresivos y antipsicóticos], así como polimedicación [definida como 5 o más fármacos]).

### Análisis estadístico

Se realizó un análisis descriptivo de las variables, expresando las cuantitativas mediante mediana y rango intercuartílico y las cualitativas mediante frecuencias y porcentajes. Para analizar los posibles factores relacionados con el MC se realizó un análisis bivariante, utilizando la χ^2^ para las variables cualitativas y la U de Mann-Whitney para las cuantitativas, tras comprobar, mediante el test de Kolmogorov-Smirnov, la ausencia de normalidad de dichas variables. Posteriormente se realizó un análisis multivariante mediante un modelo de regresión logística binaria. La selección del mejor modelo se realizó con el método paso a paso, incluyéndose todas las variables que en el análisis bivariante habían sido significativas (p ≤ 0,05). Para asegurar el buen ajuste del modelo multivariante se realizó la prueba de Hosmer y Leveshow. Se consideró como umbral de significación estadística una p < 0,05. El análisis estadístico de los resultados se realizó con el programa SPSS versión 22 para Windows (IBM©).

### Consideraciones éticas

El estudio contó con la aprobación del Comité de Ética de Investigación con Medicamentos del Hospital Gregorio Marañón y de la Comisión Local de Investigación Sur de la Gerencia Asistencial de Atención Primaria. Los pacientes que acudieron a las entrevistas fueron informados detalladamente sobre el estudio, se les entregó el consentimiento informado (CI), contestando a todas las dudas que pudieron surgir. Una vez firmado el CI se procedió a realizar las valoraciones pertinentes para llevar a cabo el estudio. La investigación no interfirió en la práctica clínica ni en el funcionamiento habitual del centro de salud.

## Resultados

En la tabla 1 se muestra la ausencia de diferencias significativas entre la población de participantes y no participantes (rechazaron participar en el estudio). La tabla 2 describe las características del total de participantes (189) y desagregado por sexo. La mediana de edad de los participantes fue de 77,0 años (73,0-80,0), siendo el rango de edad de 70 a 95 años. El 62% eran mujeres, y el 26% vivían solos/as. El 25% refirieron haberse caído en el último año. El 27% tenían alteraciones en el equilibrio y disminución de la fuerza en los miembros inferiores. El 50% tenían una velocidad de la marcha disminuida. El uso de benzodiacepinas y antidepresivos es mayor en mujeres y la comorbilidad es mayor en varones (p < 0,05).

**Tabla 1:** Características poblacionales de participantes y no participantes en el estudio

Variable	Participación de los sujetos (n = 300) (mediana o porcentaje)			
	Sí participan (n = 189)	No participan (n = 111)	p	OR (IC 95%)
Sexo hombre	38% (72)	41% (46)	0,567	0,9 (0,5-1,4)
Edad (años)	77,0 (73,0-80,0)	75,0 (73,0-79,0)	0,224	
Índice de Barthel	95,0 (95,0-100,0)	100,0 (93,8-100,0)	0,144	

Se muestran porcentajes (frecuencias) para las variables cualitativas y mediana (rango intercuartílico) para las variables cuantitativas.

**Tabla 2:** Características de los participantes

Variable	Total (n = 189)	Mujeres (n = 117)	Hombres (n = 72)	p-valor	OR (IC 95%)
Edad	77,0 (73,0-80,0)	76,0 (73,0-79,0)	77,5 (75,0-80,8)	0,081	
Índice de Barthel	95,0 (95,0-100,0)	95,0 (95,0-100,0)	100,0 (95,0-100,0)	0,266	
Vive solo/a	26% (49)	33% (38)	15% (11)	0,009*	2,7 (1,3-5,6)
Prueba de ejecución (SPPB)	11,0 (10,0-12,0)	11,0 (10,0-12,0)	11,0 (10,0-12,0)	0,099	
Alteraciones del equilibrio	27% (51)	32% (37)	19% (14)	0,067	1,9 (1,0-3,9)
Disminución en la velocidad de la marcha	50% (95)	54% (63)	44% (32)	0,209	1,5 (0,8-2,6)
Disminución de la fuerza en miembros inferiores	27% (51)	30% (35)	22% (16)	0,247	1,5 (0,8-3)
Alto riesgo de caídas (Downton ≥ 3)	39% (74)	43% (50)	33% (24)	0,198	1,5 (0,8-2,8)
Antecedentes de caídas en el último año	25% (47)	27% (32)	21% (15)	0,314	1,4 (0,7-2,9)
Lesiones asociadas a las caídas	56% (106)	65% (76)	42% (30)	0,002*	2,6 (1,4-4,7)
Tiempo desde la última caída (meses)	24,0 (4,3-59,5)	24,0 (6,0-60,0)	24,0 (1,0-58,0)	0,360	
IMC	30,2 (27,2-32,7)	30,3 (27,4-32,7)	30,0 (26,8-32,7)	0,563	
Uso de gafas	58% (110)	57% (67)	60% (43)	0,739	0,9 (0,5-1,6)
Uso de audífono	8% (15)	9% (10)	7% (5)	0,692	1,3 (0,4-3,8)
Uso de dispositivo para la marcha	19% (36)	18% (21)	21% (15)	0,624	0,8 (0,4-1,7)
Comorbilidad (índice de Charlson ≥ 3)	11% (21)	3% (4)	24% (17)	< 0,001*	0,1 (0-0,4)
Consumo de hipotensores	79% (149)	76% (89)	83% (60)	0,235	0,6 (0,3-1,3)
Consumo de benzodiacepinas	20% (38)	27% (31)	10% (7)	0,005*	3,3 (1,4-8)
Consumo de antidepresivos	19% (35)	24% (28)	10% (7)	0,015*	2,9 (1,2-7)
Polimedicación (≥ 5 fármacos)	43% (81)	38% (45)	50% (36)	0,120	0,6 (0,3-1,1)
Miedo a caerse (escala Short FES-I)	10,0 (7,0-14,0)	11,0 (8,0-15,0)	8,0 (7,0-11,0)	< 0,001*	

Se muestran porcentajes (frecuencias) para las variables cualitativas y mediana (rango intercuartílico) para las variables cuantitativas.

* p < 0,05. Significación entre ambos sexos.

La mediana de la puntuación del Short FES-I fue de 10,0 (RIC = 7,0-14,0), en varones 8 (RIC = 7,0-11,0), frente a 11 (RIC = 8,0-15,0) en mujeres (p < 0,05).

La prevalencia global del MC en la población de estudio fue del 43% (IC 95%: 35,5-50,2), siendo mayor en mujeres que en hombres (52% vs 28%; p < 0,05) y aumentando según se incrementa la edad (55% de ≥ 80 años, frente al 38% en pacientes de 70-79 años; p < 0,05) (tabla 3).

**Tabla 3:** Prevalencia del miedo a caerse desagregado por edad

Miedo a caerse	Total (n = 189)	Sí (n = 81)	No (n = 72)	p	OR (IC 95%)
Edad ≥ 80	100% (53)	55% (29)	45% (24)	0,040	2 (1-3,7)
Edad 70-80	100%(136)	38% (52)	62% (84)		
Total	100% (189)	43% (81)	57% (108)		

Se muestran porcentajes (frecuencias).

En el análisis bivariante realizado el MC está relacionado con ser mayor de 80 años, ser mujer, vivir solo, ser capaz para realizar ABVD (índice de Barthel), cribado positivo del riesgo de caídas (Downton ≥ 3), caídas en el último año, caídas previas, presencia de lesiones relacionadas con las caídas, cribado positivo de la fragilidad según la escala SPPB, alteraciones en el equilibrio, velocidad de la marcha, disminución de la fuerza en miembros inferiores, uso de dispositivo de la marcha y audífono, así como el consumo de hipotensores y antidepresivos (tabla 4).

**Tabla 4:** Factores asociados con el miedo a caer (análisis bivariante)

	Miedo a caerse (n = 189) (mediana o porcentaje)			
Variable	Sí (n = 81)	No (n = 108)	p	OR (IC 95%)
Sexo mujer	75% (61)	52% (56)	0,001*	2,8 (1,5-5,3)
Edad (años)	78,0 (73,0-82,0)	76,0 (73,0-79,0)	0,084	
Edad > 80 años	36% (29)	22% (24)	0,040*	2 (1-3,7)
Índice de Barthel	95,0 (90,0-100,0)	100,0 (95,0-100,0)	< 0,001*	
IMC	30,5 (27,3-32,8)	30,0 (27,1-32,1)	0,599	
Vivir solo	36% (29)	19% (20)	0,007*	2,5 (1,3-4,8)
Alto riesgo de caídas (Downton ≥ 3)	64% (52)	20% (22)	< 0,001*	7 (3,7-13,5)
Caídas en el último año	37% (30)	16% (17)	0,001*	3,1 (1,6-6,3)
Caídas previas	86% (70)	51% (55)	< 0,001*	6,1 (2,9-12,8)
Lesiones asociadas a caídas previas	75% (61)	42% (45)	< 0,001*	4,3 (2,3-8)
Tiempo desde última caída (meses)	18,0 (4,0-48,0)	24,0 (4,5-60,0)	0,177	
Prueba de ejecución (SPPB)	10,0 (8,0-11,0)	12,0 (11,0-12,0)	< 0,001*	
Alteraciones en el equilibrio	43% (35)	15% (16)	< 0,001*	4,4 (2,2-8,7)
Velocidad de la marcha disminuida	72% (58)	34% (37)	< 0,001*	4,8 (2,6-9)
Fuerza en miembros inferiores disminuida	44% (36)	14% (15)	< 0,001*	5 (2,5-10)
Uso de dispositivo de la marcha	32% (26)	9% (10)	< 0,001*	4,6 (2-10,3)
Comorbilidad (índice de Charlson ≥ 3)	10% (8)	12% (13)	0,640	0,8 (0,3-2)
Polimedicación (≥ 5 fármacos)	47% (38)	40% (43)	0,329	1,3 (0,7-2,4)
Uso de gafas	62% (50)	56% (60)	0,395	1,3 (0,7-2,3)
Uso de audífono	14% (11)	3,7% (4)	0,013*	4,1 (1,3-13,3)
Consumo de hipotensores	72% (58)	84% (91)	0,035*	0,5 (0,2-0,95)
Consumo de benzodiacepinas	24% (19)	18% (19)	0,320	1,4 (0,7-2,9)
Consumo de antidepresivos	27% (22)	12% (13)	0,008*	2,7 (1,3-5,8)

Se muestran porcentajes (frecuencias) para las variables cualitativas y mediana (rango intercuartílico) para las variables cuantitativas.

* p < 0,05. Significación entre miedo a caerse: sí/no.

El modelo multivariante final incluye las variables que se asociaron de manera significativa con el MC: ser mujer, vivir solo, uso de hipotensores, puntuación en SPPB (como indicador de fragilidad), riesgo de caídas positivo y lesiones asociadas a caídas previas (tabla 5). El valor de R^2^ del modelo es 0,45. Además, según los datos aportados por el programa estadístico, este modelo clasificaría correctamente al 78,3% de los casos. Concretamente, el 69,1% de los que tienen MC y el 85,2% de los que no lo tienen. La ausencia de significación (p = 0,707) en la prueba de Hosmer y Leveshow nos informa además del buen ajuste del modelo.

**Tabla 5:** Variables relacionadas con el miedo a caer. Modelo final de regresión logística

	B	p	OR ajustada	IC 95%	
Variables independientes				Inferior	Superior
Riesgo de caídas	1,167	0,003	3,2	1,5	7,0
SPPB	0,506	0,000	1,7	1,3	2,2
Consumo de hipotensores	− 0,976	0,030	0,4	0,2	0,9
Vivir solo	0,910	0,031	2,5	1,1	5,7
Lesiones asociadas a caídas previas	1,040	0,011	2,8	1,3	6,3
Constante	−6,573	0,000	0,001		

## Discusión

La prevalencia de MC obtenida en personas mayores que viven en la comunidad a través del short FES-I, del 43% (IC 95%: 35,5-50,2), está en concordancia con la estimación fijada por los investigadores al inicio y con el resto de los estudios de prevalencia en España que han tratado de identificar el MC en personas mayores que viven en la comunidad (41,5-49,4%) utilizando otros métodos de cribado^7^, ^17^, ^18^.

Si comparamos los datos obtenidos en nuestro estudio con los publicados a nivel internacional observamos que existen importantes discrepancias. Así, Rivasi et al.^26^ identificaron una prevalencia del 15,1% a través de una modificación del FES-I (MFES-I) en una población irlandesa de mayores de 60 años. Vitorino et al.^27^ obtuvieron una prevalencia del 54,1% en Portugal y del 45,9% en Brasil mediante el FES-I en una muestra de pacientes mayores de 64 años que acudían al centro de salud. En tres estudios realizados en Asia (Corea, Japón y Taiwán) las prevalencias son similares a las publicadas en este estudio, oscilando entre el 43 y el 53%^5^, ^8^, ^12^, ^21^. Sin embargo, en el estudio de Liu^20^, realizado con población mayor de 64 años de Hong Kong, se obtuvo una prevalencia del 64,3% utilizando la escala *Chinese FES-I*, si bien es cierto que el 75% de los participantes eran mujeres. Por último, señalar que en un estudio realizado en Turquía por Simsek et al.^22^ con población de muy avanzada edad (mayores de 80 años) la prevalencia obtenida también fue muy superior (86,6%). Entre las causas que pueden justificar la variabilidad encontrada estarían las distintas características de los participantes en cuanto a edad y sexo, las escalas de medida utilizadas y la cultura de cada país o región.

Los factores asociados al MC identificados en el modelo multivariante realizado coinciden con los publicados en otros estudios: ser mujer^3^, ^7^, ^17^, ^20^, ^21^, ^22^, ^23^, vivir solo^22^, baja puntuación en SPPB (como indicador de fragilidad)^7^, riesgo de caídas positivo^22^ y consumo de hipotensores, siendo este último identificado como factor protector. La relación identificada en nuestro estudio del MC con los antecedentes de lesiones asociadas a las caídas (contusiones, factura y/o hospitalización) no ha sido constatada en otras publicaciones, por lo que será necesario realizar estudios longitudinales que permitan confirmar esta posible relación causal. En cuanto al efecto protector del uso de los hipotensores, contrasta en cierta medida con la bibliografía publicada hasta la fecha^26^, ^28^, en la que los pacientes hipertensos en tratamiento farmacológico presentan un aumento significativo del MC. Esta discrepancia puede deberse a las diferentes características de las poblaciones incluidas en los distintos estudios, siendo en nuestro caso un grupo de población no frágil, independiente para la deambulación y las ABVD, en el que este tipo de tratamiento puede tener menos influencia que en población más vulnerable. Hacen falta, en cualquier caso, más estudios para analizar la relación entre la capacidad funcional, la hipertensión, el uso de hipotensores y el MC.

Respecto al resto de factores relacionados con MC identificados en el análisis bivariante, nuestros datos también están en consonancia con la bibliografía consultada: el MC es mayor en personas a partir de 80 años^3^, ^8^, ^17^, ^21^, ^23^, ^26^, con antecedentes de caídas previas^3^, ^7^, ^8^, ^17^, ^18^, ^19^, ^20^, ^21^, ^22^, ^23^, ^26^, que toman antidepresivos^17^, ^26^, con menor capacidad para ABVD^26^, ^28^, menor capacidad funcional^5^, ^7^, ^18^, ^19^, ^22^, ^26^, ^29^, que utilizan dispositivos para la marcha^18^, ^22^, ^26^, con disminución de la fuerza en los miembros inferiores^8^, ^18^, ^23^, ^26^ y con alteración del equilibrio^18^, ^23^, ^26^. Por otra parte, el uso de audífonos ha resultado un factor de riesgo novedoso para el MC. El uso de estos dispositivos, como indicador de un déficit sensorial importante, en ocasiones no corregido de manera adecuada con la utilización del audífono, puede contribuir a aumentar el miedo de las personas a caerse. Aunque hacen falta más estudios para confirmar la relación entre ambas variables, se podría considerar a estos pacientes como de mayor riesgo y ser priorizados, junto a los que presentan el resto de factores de riesgo identificados, a la hora de llevar a cabo intervenciones de carácter preventivo.

Por último, señalar que en nuestro estudio no se ha encontrado asociación entre diversos factores y el MC, que sí ha sido descrita por otros autores: polimedicación^19^, ^22^, ^26^, comorbilidad^7^, ^8^, ^17^, ^19^, ^22^, ^26^, consumo de benzodiacepinas^17^, ^26^, sedantes^17^, ^26^, antipsicóticos^17^ y uso de gafas^20^. Es especialmente significativo la falta de asociación con la presencia de comorbilidad, identificada en numerosas publicaciones y que probablemente esté relacionado con la variabilidad existente a la hora de considerar el concepto de comorbilidad en los distintos estudios. También destaca la ausencia de significación de la variable «tiempo desde la última caída». En este sentido, Jang et al.^30^ encontraron un riesgo 4 veces superior de MC en los pacientes que habían caído en los últimos 6 meses.

### Aplicabilidad y relevancia clínica

Es el primer estudio que identifica la prevalencia del MC en personas mayores independientes para la deambulación a través de un cuestionario sencillo y fácil de aplicar en el contexto de atención primaria, donde la limitación del tiempo disponible en consulta siempre es un factor de limitación importante. Además, a diferencia de lo que sucede cuando se utiliza como método de cribado una pregunta cerrada (sí/no), permite identificar si las limitaciones se pueden producir en el entorno domiciliario o al realizar actividades sociales, lo que facilita anticipar posibles cambios de comportamiento en el paciente perjudiciales para otras esferas de su vida.

La prevalencia del MC en personas mayores es elevada. Los profesionales de atención primaria deben sistematizar el cribado de este problema de salud priorizando especialmente en las personas que presentan los siguientes factores de riesgo: ser mujer, vivir solo, tener una puntuación baja en el SPPB (como indicador de fragilidad) o presentar un elevado riesgo de caídas.

Los profesionales de atención primaria deben asumir un papel fundamental en el desarrollo de estrategias de cribado (detección precoz) de este problema de salud realizando la valoración sistemática del MC en personas mayores mediante el uso normalizado de la escala Short FES-I y desarrollando intervenciones estructuradas en las personas con MC que disminuyan su preocupación y el riesgo de discapacidad, como programas de actividad física multicomponente o terapia cognitiva conductual^9^. Se debe asegurar, en cualquier caso, que estas intervenciones siempre se lleven a cabo en entornos seguros y supervisadas por familiares y cuidadores o por profesionales expertos, en caso necesario.

### Limitaciones del diseño

Se necesitan estudios longitudinales para identificar relaciones causales entre la presencia de algunos de los factores de riesgo identificados en el estudio y la aparición del MC. Los participantes del estudio fueron captados a partir de aquellos que acudían al centro de salud, por lo que es posible que la prevalencia en el conjunto de pacientes adultos mayores que viven en la comunidad esté infraestimada, ya que no se ha considerado a los pacientes con dependencia grave ni a los inmovilizados o encamados. De igual modo, al haberse obtenido los datos en un único centro de salud los resultados no se podrían extrapolar de manera generalizada a todos los centros de salud, aunque sí podrían equipararse a aquellos que presentan una estructura poblacional similar.

**Lo conocido sobre el tema**

• Uno de los principales factores de riesgo de las caídas en personas mayores que viven en la comunidad es el miedo a caer (en inglés *fear of falling* [FOF]).
• El miedo a caer también se considera un factor de riesgo independiente para el desarrollo de deterioro funcional y el inicio de la fragilidad en las personas mayores.
• No se dispone de estudios en España que evalúen la prevalencia del miedo a caer en personas ancianas, independientes para la deambulación, que viven en la comunidad, mediante la versión reducida de la escala de *Falls Efficacy Scale-Internacional* *(Short FES-I)* ni de los factores que influyen en esta situación.

**¿Qué aporta este estudio?**

• Es el primer estudio que evalúa la prevalencia del miedo a caer en la población mayor, independiente para la deambulación, que vive en la comunidad, a través de un cuestionario sencillo y fácil de aplicar en el contexto de atención primaria como es el cuestionario Short FES-I.
• Los principales factores de riesgo del miedo a caer identificados son el hecho de ser mujer, vivir solo, tener una puntuación baja en el *Short Physical Performance Battery* (SPPB) como indicador de fragilidad y el riesgo de caídas positivo.
• Nuestro estudio añade evidencia sobre la necesidad de incorporar en las consultas de atención primaria intervenciones para detectar precozmente el miedo a caer en la población anciana y diseñar estrategias para abordar los factores de riesgo relacionados.

## Financiación

El coste de la publicación del artículo ha sido financiado por la Fundación para la Investigación e Innovación Biomédica de Atención Primaria de la Comunidad de Madrid (España) a través de la Política de financiación de publicaciones científicas del año 2020.

## Conflicto de intereses

Los autores declaran no tener ningún conflicto de intereses.
